# ANCA vasculitis in a patient with Alport syndrome: a difficult diagnosis but a treatable disease!

**DOI:** 10.1186/s12882-017-0527-4

**Published:** 2017-03-29

**Authors:** Valentine Gillion, Michel Jadoul, Selda Aydin, Nathalie Godefroid

**Affiliations:** 1 Division of Nephrology, Cliniques Universitaires Saint-Luc, Université Catholique de Louvain, Brussels, Belgium; 2 Division of Pathology, Cliniques Universitaires Saint-Luc, Université Catholique de Louvain, Brussels, Belgium; 3 Division of Pediatric Nephrology, Cliniques Universitaires Saint-Luc, Université Catholique de Louvain, Brussels, Belgium

**Keywords:** Alport syndrome, ANCA-associated vasculitis, Glomerulonephritis

## Abstract

**Background:**

Alport syndrome and ANCA-associated vasculitis are both rare diseases. The co-existence of these two conditions has never been reported. There is no obvious pathogenic link between these two glomerular diseases. The management of this case highlights the importance of a systematic approach when investigating the unexpected unfavourable evolution of a known glomerulopathy.

**Case presentation:**

A-17 year old caucasian boy with a genetically proven X-linked Alport syndrome presented with progressive dyspnea, fatigue and pallor. His blood tests showed a severe anemia (Hb 6.9 g/dl) with acute worsening of kidney function (serum creatinine, normal 9 months earlier, was now 3.6 mg/dl). Microscopic hematuria and proteinuria also worsened. He soon developed signs of alveolar hemorrhage. Serological tests showed the presence of perinuclear ANCA with anti MPO specificity. Kidney biopsy showed a necrotizing and crescentic glomerulonephritis. Pulses of methylprednisolone were given in combination with plasmapheresis. The patient further received 6 pulses of cyclophosphamide, followed by maintenance oral azathioprine. During the 15-months follow up he remained well with serum creatinine back to normal, and some residual proteinuria and hematuria ascribed to Alport syndrome.

**Conclusion:**

We report a young patient with the coexistence of Alport syndrome and ANCA associated vasculitis. Clinicians should be aware of the possibility of a second acquired disease in a patient with a known kidney disease, genetic in this case. This coexistence is very rare, but should be considered even if both diseases are rare, if the evolution is atypical for the single (known) primary disease. The diagnosis of the added vasculitis prompted in our case the initiation of immunosuppressive drugs, with a favourable outcome.

## Background

Alport syndrome is clinically characterized by hematuria and inconstant renal failure, hearing loss, lenticonus, and retinal flecks. Electron microscopy shows a lamellated glomerular basement membrane as a result of abnormal collagen IV composition [1]. Genetically, classically eighty-five percent of families indeed have X-linked inheritance with mutations in COL4A5, whereas the others have autosomal recessive disease with homozygous or compound heterozygous mutations in COL4A3 or COL4A4. A rarer autosomal dominant inheritance has been described more recently and results from heterozygous COL4A3 [2] or COL4A4 variants [3]. However, more recent studies have shown that autosomal forms are more frequent that initially suspected and may represent up to 30% of all cases of Alport syndrome [4–6].

**Fig. 1:**
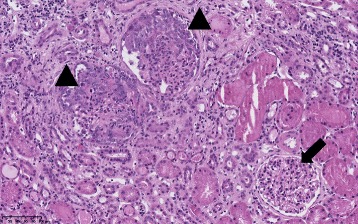
A glomerulus (*Arrow*) with no alterations and two glomeruli (*Arrowheads*) showing cellular crescents in Bowman’s space (Hematoxylin and eosin, ×191)

Only a few cases of Alport syndrome associated with other glomerular diseases have been reported. In a review of double glomerulopathy cases in children, Cheong and colleagues [7] described three cases of pediatric patients with Alport syndrome and post infectious glomerulonephritis and one case of Alport syndrome and IgA nephropathy. The most common association occurs in kidney transplant recipients with post transplantation antiGBM disease. This disease represents an allogeneic response to the normal triple α3α4α5chains present in the graft but not in the native kidneys of the Alport recipient [8]. The initial prevalence was 3–5% but it seems rarer nowadays [9].

We report the development of severe ANCA vasculitis in an adolescent with already known X-linked Alport syndrome and discuss the diagnostic pitfalls of this rare association.

## Case presentation

A 17 year old patient was followed at the Pediatric Department for X-linked Alport syndrome since the age of 3. He presented initially with gross hematuria after an upper respiratory tract infection. A renal biopsy performed at the age of 4 made the diagnosis of Alport syndrome with 44 glomeruli showing enlargements of the mesangial stalks. Immunofluorescence showed mesangials deposits of IgG. Electron microscopy showed splitting and lamellation of the lamina densa, typical features of Alport syndrome. The diagnosis was confirmed by the detection of a new mutation in COL4A5 (c.3781G > A) considered as pathogenic. Indeed, glomerular basement membrane is made of trimers of collagen IV chains (alpha 5, alpha 4 and alpha 3). Each chain has an intermediate collagenous domain with repeated sequences of amino acids (Gly-X-Y) that play a role in the conformation of the triple helix formation [10] Missense mutations, as found in our patient, that result in glycine substitutions are the most frequent mutations described in Alport syndrome [11]. The amino acid affected by the mutation is highly conserved across species, confirming that it is a mutation rather than a polymorphism. This genetic diagnosis was made in a laboratory with extensive experience in the genetics of Alport syndrome [5].

At age 10, sensorineural deafness was detected at school. At age 11, mild proteinuria (0.55 g/24 h) was detected for the first time and lisinopril 2.5 mg was introduced. At age of 15, proteinuria increased (0.8 g/24 h) despite Lisinopril 5 mg; valsartan and hydrochlorothiazide were added [12, 13]. In April 2012, at age of 16, estimated glomerular filtration rate was 112.5 ml/min/1.73 m2 (Schwartz equation) and urinary protein/creatinine ratio was 0.185 g/g with 300 RBC/microliter.

In January 2013, he had progressive dyspnea without fever. He was prescribed a course of amoxycilline-clavulanate by his general practitioner for a presumed tracheitis, with no improvement. Two weeks later, amoxycilline-clavulanate was replaced by erythromycin. One day later, he had diarrhea with progressive weakness and he was admitted to our hospital. On physical examination, the patient was pale. Temperature was 36.3 °C; heart rate was 83 beats per minute; blood pressure was 114/85 mm Hg; respiratory rate was 17 breaths per minute; and oxygen saturation was 99% while breathing ambient air. Blood tests showed: serum creatinine level 3.60 mg per deciliter (316.8 μmol per liter); urea 210 mg per deciliter (74.4 mmol per liter), haemoglobin 6.9 g per deciliter, WBC 12900 per cubic millimeter, platelets 401,000 per cubic millimeter. The C reactive protein serum level was 3.3 mg/dl (normal < 1 mg/dl). Urinalysis showed 200 RBC per microliter with a urinary protein/creatinine ratio of 0.66 g/g. Chest X-ray as well as kidney ultrasonography were unremarkable. The acute renal failure was initially ascribed to dehydration combined with dual renin-angiotensin system blockade. These drugs were stopped. The cause of the anemia was however unclear. Packed red cell transfusions and saline were given during two days. Anemia persisted: a bone marrow biopsy was performed, that proved normal. After 3 days of intravenous saline, serum creatinine plateaued around 1.87 mg per deciliter (164.5 μmol per liter), with worsening proteinuria (1 g/g) and hematuria (1000 RBC/microliter). C reactive protein level was 11.7 mg per deciliter. As the urinary protein/creatinine ratio had increased, renal failure had only partially reversed, anemia remained unexplained, and CRP had increased further, auto-immune serological tests were done on the 7th hospital day. The results were obtained within 3 days and showed the presence of P-ANCA (titer 1/1280) with > 134 AU anti-MPO by ELISA (Nl < 3.5). On the 10th hospital day, he developed an acute dyspnea with a chest X-ray suggestive of intraalveolar hemorrhage. A kidney biopsy showed necrotizing and crescentic glomerulonephritis involving 2 out of 3 glomeruli (Fig. 1). On immunofluorescence, there was no IgG and C1q fixation and mild Ig M and C3 fixation. Immunostaining for α-3 and α-5 chains of collagen IV was negative, in line with the diagnosis of Alport’s syndrome. There was no history of exposure to any environmental risk factors for developing ANCA vasculitis such as silica dust [14].

The patient received three days of pulse methylprednisolone 1000 mg IV, in combination with plasmapheresis, daily during the first week and 3 times per week during the second week. Methylprednisolone per os was subsequently given (0.75 mg/kg with progressive tapering). Serum creatinine peaked at 3.02 mg/dl on the 14^th^ hospital day, then improved rapidly (18^th^ day : 1.52 mg:dl, 22^nd^ day: 1.27 mg/dl).In addition, IV cyclophosphamide 750 mg was given every three weeks for a total of 6 doses, followed by azathioprine 150 mg as maintenance therapy.

At last follow up in July 2015, the patient was still under 150 mg azathioprine. Methylprednisolone has been discontinued in March 2014. Serum creatinine was 1.2 mg per deciliter (115 μmol per liter). ANCA were no longer detected by indirect IF whereas the anti MPO level remained slightly positive at 4.7 AU (*N* < 3.5 AU) by ELISA. Urinalysis showed moderate persistent proteinuria (protein/creatinine ratio of 0.660 g/g) and hematuria (110 RBC/microliter).

## Discussion

The prevalence of Alport syndrome is around 1 in 50,000 live births [15]. ANCA-associated vasculitis is an even much less common in childhood with an estimated annual incidence of less than 1 per 1,000,000 children [16]. The association of these two glomerular diseases has never been reported, to the best of our knowledge, and may be a coincidence, although the low incidence of both diseases theoretically would argue against a coincidence. Studies have shown an association between HLA-DQ polymorphisms and anti-myeloperoxidase ANCA vasculitis [17] but there is as yet no gene predisposition to ANCA vasculitis linked to the X chromosome, where the locus of Alport syndrome is located [18]. We speculate thus that there is no pathogenic link between these 2 diseases and the association is probably coincidental.

This uncommon association delayed routine serological testing for auto-immune glomerulonephritis in a patient with an X-linked Alport syndrome. Indeed, making the diagnosis of a new, second glomerulonephritis in patients with a known glomerular disease can be tricky. We were indeed not very concerned by the proteinuria and the microscopic hematuria since the patient was known to have an Alport syndrome. On admission, the anemia was thought to be secondary to inflammation. Eventually, the increase of the urinary protein/creatinine ratio, admittedly multifactorial (Renin Angiotensin System blockade had been stopped), the poor reversibility of renal failure, the persistent inflammation and the unexplained anemia made us suspect an additional disease. Indeed, the initial diagnosis (Alport Syndrome), although firmly established, did not account easily for many recent findings: we avoided thus the not infrequent cognitive error in medical diagnosis, called premature closure (or reluctance to pursue alternative diagnoses once a commitment is made) [19].

The search for ANCA and anti GBM antibodies led to the surprising diagnosis of an ANCA-associated vasculitis. Soon after this diagnosis, the patient developed intra-alveolar haemorrhage, a classical feature of ANCA-vasculitis.

## Conclusions

 This case highlights the importance of being systematic in case of unusual evolution of a known glomerulopathy, genetic in this case. A second type of acquired GN may be, very infrequently, present and should be diagnosed as early as possible, in order to start the appropriate drug regimen and alter the initially unfavourable course of the disease, as shown in our case.

## Abbreviations

ANCA: Anti-neutrophil cytoplasmic antibody
AU: Arbitrary unit
COL4A3: Alpha 3 chain of collagen type IV
COL4A4: Alpha 4 chain of collagen type IV
COL4A5: Alpha 5 chain of collagen type IV
CRP: C reactive protein
ELISA: Enzyme-linked immunosorbent assay
GBM: Glomerular basement membrane
GN: Glomerulonephritis
Hb: Hemoglobin
HLA: Human leukocyte antigenIF: immunofluorescence
IV: Intravenous
MPO: Myeloperoxidase
N: Normale range
P-ANCA: Perinuclear anti-neutrophil cytoplasmic antibody
RBC: Red blood cells
WBC: White blood cells
